# Overexpression of Endothelin 1 Triggers Hepatocarcinogenesis in Zebrafish and Promotes Cell Proliferation and Migration through the AKT Pathway

**DOI:** 10.1371/journal.pone.0085318

**Published:** 2014-01-08

**Authors:** Jeng-Wei Lu, Chung-Yi Liao, Wan-Yu Yang, Yueh-Min Lin, Shiow-Lian Catherine Jin, Horng-Dar Wang, Chiou-Hwa Yuh

**Affiliations:** 1 Institute of Molecular and Genomic Medicine, National Health Research Institutes, Zhunan, Miaoli, Taiwan; 2 Department of Life Sciences, National Central University, Jhongli City, Taoyuan, Taiwan; 3 Department of Pathology, Changhua Christian Hospital, Changhua City, Changhua County, Taiwan; 4 Department of Medical Technology, Jen-Teh Junior College of Medicine, Nursing and Management, Miaoli, Taiwan; 5 Institute of Biotechnology, National Tsing Hua University, Hsinchu, Taiwan; 6 Institute of Bioinformatics and Structural Biology, National Tsing Hua University, Hsinchu, Taiwan; 7 Department of Biological Science and Technology, National Chiao Tung University, Hsinchu, Taiwan; National University of Singapore, Singapore

## Abstract

Hepatocarcinogenesis commonly involves the gradual progression from hepatitis to fibrosis and cirrhosis, and ultimately to hepatocellular carcinoma (HCC). Endothelin 1 (*Edn1*) has been identified as a gene that is significantly up-regulated in HBx-induced HCC in mice. In this study, we further investigated the role of *edn1* in hepatocarcinogenesis using a transgenic zebrafish model and a cell culture system. Liver-specific *edn1* expression caused steatosis, fibrosis, glycogen accumulation, bile duct dilation, hyperplasia, and HCC in zebrafish. Overexpression of *EDN1* in 293T cells enhanced cell proliferation and cell migration in *in vitro* and xenotransplantation assays and was accompanied with up-regulation of several cell cycle/proliferation- and migration-specific genes. Furthermore, expression of the unfolded protein response (UPR) pathway-related mediators, such as spliced XBP1, ATF6, IRE1, and PERK, was also up-regulated at both the RNA and protein levels. In the presence of an EDN1 inhibitor or an AKT inhibitor, these increases were diminished and the EDN1-induced migration ability also was disappeared, suggesting that the EDN1 effects act through activation of the AKT pathway to enhance the UPR and subsequently activate the expression of downstream genes. Additionally, p-AKT is enhanced in the *edn1* transgenic fish compared to the GFP-mCherry control. The micro RNA miR-1 was found to inhibit the expression of EDN1. We also observed an inverse correlation between EDN1 and miR-1 expression in HCC patients. In conclusion, our data suggest that EDN1 plays an important role in HCC progression by activating the PI3K/AKT pathway and is regulated by miR-1.

## Introduction

Hepatocarcinogenesis involves the gradual progression from hepatitis to fibrosis and cirrhosis, and ultimately to hepatocellular carcinoma (HCC). HCC is the most common type of liver cancer and has a poor prognosis, especially in Asia and Africa [1]. Factors associated with an increased risk of HCC include viral infection by hepatitis B virus (HBV) or hepatitis C virus HCV (HCV), chronic alcohol consumption, tobacco smoking, cirrhosis, and aflatoxin [2]–[6]. The multistep process of HCC is initiated by hyperplasia, which is followed by dysplasia, early HCC, and finally, full-blown HCC [7]. The therapeutic options for advanced or metastatic HCC are very limited [8], partially because the molecular mechanisms underlying hepatocarcinogenesis remain unclear [9]. Suitable animal models and efficient cell culture systems have provided a major breakthrough for understanding these mechanisms [10], [11].

Endothelin 1 (EDN1) is a 21-amino acid peptide that exerts a wide range of biological activities. It is recognized as a vasoconstrictor peptide and has been implicated in the growth regulation of several tumors [12], [13]. EDN1 has been found to enhance tumor growth by promoting angiogenesis [14], [15]. Vascular endothelial growth factor (VEGF) has been shown to enhance the expression and secretion of EDN1 in endothelial cells [16]. Previous studies have revealed that EDN1 may also affect tumor invasion and metastasis [17], [18]. The increased expression of EDN1 has been observed in many malignant tumors, including breast, colorectal, prostate, pancreatic, and hepatocellular carcinomas [19]–[25].

EDN1 secretion in hepatoma cells has been described [26], [27]. Hepatoma cell growth increases upon the exogenous addition of EDN1 in a dose-dependent manner [12]. The tissue concentrations of EDN1, big ET-1, and the ETA receptor were significantly increased in hepatoma tissue compared to normal liver tissue [12]. Previously, in a hepatitis B virus X antigen-induced HCC mouse model, we identified genes that were significantly up-regulated at the pre-cancer and cancer stages, including Edn1, Src, Bmp4, and Bmp7 [28]. Transient transfection of the *EDN1* gene into Hep3B cells increases cell viability, promotes cell migration and invasiveness, and enhances colony formation of these cells [28]. In addition, it has been reported that methylation-mediated silencing of the miR-1 gene induces hepatoma cell proliferation [29]. miR-1 inhibits *EDN1* expression and leads to attenuation of hepatoma cell proliferation [28]. These results suggest that a decrease of miR-1-mediated repression of *EDN1* may contribute to the development of HCC.

The zebrafish is an excellent animal model for studying liver disease and HCC. Many zebrafish disease models are similar to human disease states with respect to morphology and the expression profiles of dysregulated genes [30], [31]. Previously, we found that HBx and aflatoxin B1 act synergistically to cause hepatitis, steatosis, and liver hyperplasia [32], and overexpression of HBx together with p53 mutation caused HCC [32]. Co-expression of HBx and the HCV core protein in liver tissues induces intrahepatic cholangiocarcinoma in zebrafish [33]. Additionally, the liver-specific expression of Kras (V16) reportedly induces HCC in zebrafish [34], [35]. Furthermore, the inducible overexpression of xmrk and Myc also causes HCC; however, the HCC regresses rapidly upon withdrawal of the inducer [33], [36]. The aim of the present study was to identify the role of *edn1* in promoting liver disease and tumorigenesis and to understand the underlying mechanism using a zebrafish model and an *in vitro* cell culture system.

In this report, we demonstrate that the liver-specific overexpression of *edn1* results in HCC. Additionally, we show that a cell line stably overexpressing *EDN1* exhibits enhanced migration, proliferation, and invasion, and these effects can be reversed by the presence of EDN1 inhibitor or AKT inhibitor.

## Materials and Methods

### Zebrafish husbandry

The zebrafish embryos, larvae, and adult fish were maintained in the Zebrafish Core Facility at NTHU-NHRI (ZeTH) according to established protocols and methods [37]. The animal protocols for zebrafish experiments were approved by the Institutional Animal Care Use Committee (IACUC) of the National Health Research Institutes. The animal protocol numbers are NHRI-IACUC-100062-A and NHRI-IACUC-101005-A.

### Generation and maintenance of transgenic zebrafish

The transgenic founders were created using the Tol2 transposon system as described previously [38]. The construction of the Tol2 construct, which contains *edn1* under the control of *fabp10a*, was described previously [32]. The zebrafish *edn1* gene was amplified using the attB1-*edn1*-F and attB2-*edn1*-R primers and the 24-hr zebrafish embryo cDNA as a template. The primer sequences are listed in Table 1.

**Table 1:** Oligonucleotides primers used in this study.

Gene Name	Primer name	Start end	Sequence (5′ to 3′)	Accession number	Size (bp)
*edn1*	attB1-F-*edn1*	1	GGGGACAAGTTTGTACAAAAAAGCAGGCT ATGCATTTGAGGATTATTTTCCCAGTTCTGACC	NM_131519.1	567
	attB2-R-*edn1*	567	GGGGACCACTTTGTACAAGAAAGCTGGGT CTATGAGTTTTCAGAAATCCACGCTTGGC		
*srebf1*	Q-*srebf1*-F	2163	CATCCACATGGCTCTGAGTG	NM_001105129.1	250
	Q-*srebf1*-R	2412	CTCATCCACAAAGAAGCGGT		
*cebpa*	Q- *cebpa*-F	1080	AACGGAGCGAGCTTGACTT	NM_131885	250
	Q- *cebpa*-R	1329	AAATCATGCCCATTAGCTGC		
*fasn*	Q-*fasn*-F	7183	ATCTGTTCCTGTTCGATGGC	XM_682295	250
	Q-*fasn*-R	7432	AGCATATCTCGGCTGACGTT		
*agapt*	Q*-agapt*-F	517	TTGGCGAAAAAGGAACTGTC	NM_212992	250
	Q-*agapt*-R	820	GGTGGTACTTGAGTTTGGGG		
*caveolin*	Q-*caveolin* -F	1832	AGCAGGTCGTGAAGATTGAA	BC115201.1	250
	Q-*caveolin* -R	2081	CCACTTTAGCAGCAGCCTCT		
*ccna1*	Q-*ccna1*-F	151	TTGTGCTTGGTGTTTTGACC	NM_212818.1	197
	Q-*ccna1*-R	347	TAGCAGTTCTGAAGGCAGCA		
*ccnb1*	Q-*ccnb1*-F	603	GCGTGCCATTCTTATCGACT	NM_131513.1	199
	Q-*ccnb1*-R	801	TGCAATCTCTGGTGGGTACA		
*ccne1*	Q-*ccne1*-F	371	TCCCGACACAGGTTACACAA	NM_130995.1	201
	Q-*ccne1*-R	571	TTGTCTTTTCCGAGCAGGTT		
*ccng1*	Q-*ccng1*-F	603	GCTCAACTGGAAGGTCAAGG	NM_199481.1	199
	Q-*ccng1*-R	801	CAGGGCCAGAAGAGACAAAG		
*cdk1*	Q-*cdk1*-F	779	CTCTGGGGACCCCTAACAAT	NM_212564.2	200
	Q-*cdk1*-R	978	CGGATGTGTCATTGCTTGTC		
*cdk2*	Q-*cdk2*-F	794	CAGCTCTTCCGGATATTTCG	NM_213406.1	199
	Q-*cdk2*-R	992	CCGAGATCCTCTTGTTTGGA		
*pcna*	Q-*pcna*-F	571	GGCAACATCAAGCTCTCACA	NM_131404.1	195
	Q-*pcna*-R	765	TGCAATTTTGTCCTCAACCA		
*col1a1a*	Q-*col1a1a*-F	2811	TATTGGTGGTCAGCGTGGTA	NM_199214.1	199
	Q-*col1a1a*-R	3009	TCCTGGAGTACCCTCACGAC		
*ctgfa*	Q-*ctgfa*-F	481	TGTGTGTTTGGTGGAATGGT	NM_001015041.2	198
	Q-*ctgfa*-R	678	GGAGTCACACACCCACTCCT		
*hpse*	Q-*hpse*-F	713	GCTCTGGTTTGGAGCTCATC	NM_001045005.1	203
	Q-*hpse*-R	915	GAAATCCCGACCAAGTTGAA		
*lepr*	Q-*lepr*-F	1471	AAACGCCCCTCTTTACCTGT	NM_001113376.1	204
	Q-*lepr*-R	1674	GCTCCAGTCGCTCCAGTATC		
*mmp2*	Q-*mmp2*-F	191	TCTTGCTTCCCTGCAAACTT	NM_198067.1	209
	Q-*mmp2*-R	399	GGTCAATCTCCCCTGTCTCA		
*tgfb1a*	Q-*tgfb1a*-F	1027	TTTCGGAAAGATCTGGGTTG	NM_182873.1	199
	Q-*tgfb1a*-R	1225	AAAGAATTGGCAGAGGGTCA		
*timp2a*	Q-*timp2a*-F	327	CGTTCTGCAATGCTGATGTT	NM_182874.1	202
	Q-*timp2a*-R	528	TCCAAATTGGTCACTCCACA		
*tp53*	Q-*p53*-F	597	TTGTCCCATATGAAGCACCA	NM_131327.1	200
	Q-*p53*-R	796	TTTCCTGTCTCTGCCTGGAC		
*mycb*	Q*-mycb-F*	688	GGTGTTTCCCTTTCCACTGA	NM_200172.1	197
	Q*-mycb-R*	884	TTCTCTTTTCCACCGTGACC		
*ccnd1*	Q-*ccnd1*-F	769	TTCCTTGCCAAACTGCCTAT	NM_131025.4	201
	Q-*ccnd1*-R	969	GGTGAGGTTCTGGGATGAGA		
*edn1*	Q-*edn1*-F	220	GGAAACGCTCCACGTAAGAA	NM_131519.1	208
	Q-*edn1*-R	427	TTTCTGCCAGCTTGTGTTTG		
*18s*	Q-*18s*-F2	551	GAGAAACGGCTACCACATCC	XM_001922869.1	169
	Q-*18s*-R2	719	ACCAGACTTGCCCTCCAA		
*actin*	Q-*actin* -F	893	CTCCATCATGAAGTGCGACGT	NM_131031.1	180
	Q-*actin* -R	1072	CAGACGGAGTATTTGCGCTCA		
*EDN1*	Q-*EDN1*-F	367	TGCCAAGCAGGAAAAGAACT	NM_001955.4	195
	Q-*EDN1-*R	561	TTTGACGCTGTTTCTCATGG		
*18S*	Q-*18S*-F	1332	ATGGCCGTTCTTAGTTGGTG	M10098.1	217
	Q-*18S*-R	1548	CGCTGAGCCAGTCAGTGTAG		
*AKT3*	Q-*AKT3*-F	954	AGAAGATAATGACTATGGCCG	NM_005465.4	100
	Q-*AKT3*R	1052	ATGGTCCTGGTTGTAGAAAG		
*OS9*	Q-*OS9*-F	900	CCCGACCAAGGATGACA	NM_006812.3	140
	Q-*OS9-*R	1039	GAGCACCAGAAGCTGAAT		
*CCNA2*	Q-*CCNA2*-F	1061	TTATTGCTGGAGCTGCCTTT	NM_001237.3	224
	Q-*CCNA2-*R	1284	CTCTGGTGGGTTGAGGAGAG		
*CCNE2*	Q-*CCNE2*-F	1086	CTATTTGGCTATGCTGGAGG	NM_057749.2	101
	Q-*CCNE2-*R	1186	TCTTCGGTGGTGTCATAATG		
*CDKN2B*	Q-*CDKN2B*-F	77	TAGTGGAGAAGGTGCGACAG	NM_004936.3	166
	Q-*CDKN2B-*R	242	GTGAGAGTGGCAGGGTCTG		
*PCNA*	Q-*PCNA*-F	161	CTGAGGGCTTCGACACCTAC	NM_004530.4	170
	Q-*PCNA-*R	330	TTTCTCCTGGTTTGGTGCTT		
*MMP2*	Q-*MMP2*-F	417	AGTGGATGATGCCTTTGCTC	NM_004530.4	154
	Q-*MMP2-*R	570	GAGTCCGTCCTTACCGTCAA		
*MMP9*	Q-*MMP9-*F	1144	GACAAGAAGTGGGGCTTCTG	NM_004994.2	171
	Q-*MMP9-*R	1314	GCCATTCACGTCGTCCTTAT		
*ATF6*	Q-*ATF6-*F	546	GCCTTTATTGCTTCCAGCAG	NM_007348.3	165
	Q-*ATF6-*R	710	TGAGACAGCAAAACCGTCTG		
*BIP*	Q-*BIP-*F	1541	AAGACAAGGGTACAGGGA	NM_005347.4	106
	Q-*BIP-*R	1646	GCAAACTTCTCAGCATCATTAAC		
*IRE1*	Q-*IRE1-*F	1868	CGAACGTGATCCGCTAC	AF059198.1	101
	Q-*IRE1-*R	1968	CTTCTGCTCCACATACTCTTG		
*PERK*	Q-*PERK-*F	1643	CTCACAGGCAAAGGAAGGAG	NM_004836.5	179
	Q-*PERK-*R	1821	AACAACTCCAAAGCCACCAC		
*XBP1(total)*	Q-*XBP1(t)-*F	393	GGAGTTAAGACAGCGCTTGG	NM_005080.3	248
	Q-*XBP1-*R	640	ACTGGGTCCAAGTTGTCCAG		
*XBP1(unspliced)*	Q-*XBP1(un)-*F	499	CTCAGACTACGTGCACCTCT GCAGCA	NM_005080.3	142
	Q-*XBP1-*R	640	ACTGGGTCCAAGTTGTCCAG		

### Collection of liver tissue and histochemical analysis

A total of 37 samples were collected from the *edn1* transgenic fish at five different ages (3, 5, 7, 9, and 11 months) as described previously [32]. The histochemistry and immunohistochemistry analyses were conducted as described previously [32]. The multiple cancer tissue array (catalog #: MC801) and multiple normal tissue array (catalog #: BN1002a) were purchased from US Biomax, Inc. (Rockville, MD, USA) for the endothelin 1 immunohistochemistry analysis. The Liver cancer survey tissue array (catalog#: LV809) were purchased from US Biomax (Rockville, MD, USA) for miR-1 *in situ* hybridization and EDN1 immunohistochemistry analysis. The staining intensity of EDN1 was scored as 0 (<5%), 1 (5–25%), 2 (25%–50%), or 3 (50%–100%) based on the percentage of positively stained cells as described previously [28], [32]. Diagnoses of the Edn1 transgenic fish liver tissue were determined by a single-blind evaluation of all samples by a trained pathologist (Dr. Yueh-Min Lin, Changhua Christian Hospital, Changhua, Taiwan).

### Immunohistochemistry analyses

For immunohistochemistry, the sections were incubated at 4°C overnight with 1∶100 dilutions of primary antibodies, including rabbit anti-active caspase 3 (BD Biosciences, San Jose, CA, USA), mouse anti-PCNA (Santa Cruz Biotechnology, Inc., Santa Cruz, CA, USA), rabbit anti-endothelin 1 (GeneTex Inc., Hsinchu, Taiwan), rabbit anti-phospho-AKT (Ser473) (Cell Signaling Technology, Danvers, MA, USA), and anti-AKT (Cell Signaling Technology, Danvers, MA, USA). After washing with PBS, the sections were incubated with a 1∶100 dilution of the secondary antibody, either goat anti-rabbit IgG (Santa Cruz Biotechnology, Inc., Santa Cruz, CA, USA) or goat anti-mouse IgG (Santa Cruz Biotechnology, Inc. Santa Cruz, CA, USA), at room temperature. The protocol of immunohistochemistry was described previously [32].

### *In situ* hybridization of miR-1

*In situ* hybridization of miR-1 was performed using the miRCURY LNA microRNA ISH Optimization Kit (Exiqon, Vedbaek, Denmark). Locked nucleic acid (LNA)-modified DNA oligonucleotide probe (hsa-miR-1, ACATACTTCTTTACATTCCA; Exiqon, Vedbaek, Denmark) were used to detect the *in situ* hybridization signals of miR-1 in liver tissue sections. The tissue sections were then dehydrated, cleared, mounted, and examined by light or fluorescence microscopy.

### Sirius Red, Periodic Acid-Schiff, TUNEL, and oil red O staining

All of the staining procedures were performed as described previously [32]. The scoring method was also described previously [32].

### RNA isolation and quantitative RT-PCR

Liver tissues were frozen in liquid nitrogen immediately after dissection and stored at -70°C till RNA extraction. RNA isolation and qPCR were performed as described previously [32]. The qPCR primer sequences are listed in Table 1.

### Cell lines

The 293T cell line was obtained from the Bioresource Collection and Research Center (Taiwan). The 293T cells were cultured at 37°C with 5% CO_2_ in Dulbecco's modified Eagle's medium (DMEM) (Gibco, Grand Island, NY, USA) supplemented with 10% fetal bovine serum (Gibco) and 1% penicillin-streptomycin (Gibco).

### Expression constructs and transfection

To generate 293T cells with stable EDN1 expression, two expression constructs, pDsRed-Monomer-Hyg-N1:*EDN1* and pDsRed-Monomer-Hyg-N1, were generated and used to transfect 293T cells. *EDN1* was amplified using a human cDNA derived from the Hep3B hepatoma cell line as a template and the following primers: SalI-F-h*EDN1*, 5′ GATCGTCGAC **ATG**GATTATTTGCTCATGATTTTCTC 3′ (the *Sal*I site is underlined, and the ATG translation initiation site of EDN1 is bolded) and *BamH*I-R-h*EDN1*, ^5′^GATCGGATCC **CA**CCAATGTGCTCGGTTGTG^3′^ (the *BamH*I site is underlined. CA is the reverse complement of TG, which is the first two nucleotides of the TGA stop codon and is bolded). The amplified product was cloned into the *Sal*I and *BamH*I sites of the pDsRed-Monomer-Hyg-N1 vector. The cells were transfected with this expression construct using the Mirus TransIT®-LT1 Transfection Reagent (Mirus Bio LLC, Madison, WI, USA) according to the manufacturer's instructions. Briefly, 293T cells were seeded at 3×10^6^ cells in 100-mm dish and grown for 18 to 24 hours until they achieved 50%∼70% confluence. Serum-free DMEM medium was pre-mixed with 15 µg of plasmid and 45 µl of TransIT-LT1 reagent (1.5 ml final volume), incubated at room temperature for 25 minutes, and then added to cells with 15.5 ml complete medium. The stable clones were selected in the presence of 300 µg/ml hygromycin for 2 weeks.

### Cell proliferation assay

Cell proliferation was analyzed by the WST-1 assay. Cells (6×10^3^) were seeded in 96-well plates and each well contained 100 µl of complete DMEM medium. After 18 hours of incubation, the media was removed and 100 µl of fresh complete DMDM medium containing premixed WST-1 cell proliferation reagent (Clontech, Mountain View, CA, USA) (1∶10 dilution in complete DMEM medium) was added to each well. The plates were incubated at 37°C with 5% CO_2_ for 3 hours, followed by shaking thoroughly for 1 minute on a shaker. The absorbance at 440 nm was measured using a microplate reader. This assay was performed each day during the 5-day experiment to calculate the growth curve.

### *In vitro* cell migration assay

Migration assays were performed in Transwell plates (BD, Franklin Lakes, NJ, USA). Initially, 1×10^5^ cells (diluted in 500 µl serum-free DMEM medium) were seeded in the upper chamber, and 1ml of complete DMEM medium was added to the lower chamber. After 18 or 48 hours, the non-migrating cells in the upper chamber were removed with a cotton swab, and the cells that had migrated through the 8-µm membrane were stained with 1X DAPI and counted. The average number of cells was calculated from ten randomly captured images (200X), and this value corresponds to the cell migration of each sample. The images were captured with a Leica DMIRB inverted fluorescence microscope coupled to a CoolSNAPTM Cooled CCD camera (Roper Scientific-Princeton Instruments, Trenton, NJ, USA).

### Xenotransplantation assay

The *fli1:EGFP* transgenic *()* zebrafish were purchased from ZIRC (Oregon, USA). At two days post-fertilization (dpf), the zebrafish embryos were dechorionated and subsequently anesthetized with tricaine (0.04 mg/ml, Sigma, St. Louis, MO, USA). 293T cells stably overexpressing EDN1 or DsRed control were harvested, diluted to a concentration of 9×10^7^ cells/ml in serum-free culture medium, and labeled with CM-Dil (red fluorescence) (Vybrant; Invitrogen, Carlsbad, CA, USA). Approximately 400 cells (4.6 nl) were implanted into the yolk of each 2-dpf embryo using a Nanoject II Auto-Nanoliter Injector (Drummond Scientific, Broomall, PA, USA). After injection, the zebrafish embryos were washed once with fish water and incubated for 1 hour at 28°C. The embryos were checked for fluorescent cells at 2 hours post-transplantation and were examined for metastasis every other day by fluorescence microscopy.

### Inhibition assay

293T cells (2×10^6^ per 10 cm^2^ dish) stably overexpressing EDN1 (passage 15) or DsRed control cells (passage 17) were seeded in dishes supplemented with DMEM medium. Dishes were cultured overnight at 37°C with 5% CO_2_ prior to the addition of the EDN1 inhibitor Ambrisentan (10 µM), the AKT inhibitor MK-2206 (0.5 µM), or the PI3K inhibitor LY294002 (25 µM)Following 48 hour treatment, cells were analyzed for RNA and protein expression levels.

### Western blot analysis

Cells were harvested and cell lysate were prepared by incubating the cells in RIPA (radio-immunoprecipitation assay) buffer containing protease inhibitor cocktail (10 µl/ml) (Roche Molecular Biochemicals, Mannheim, Germany) for 30 minutes on ice. Total protein concentrations were determined using the Bradford protein assay (Bio-Rad, Hercules, CA, USA). Cell lysate (20 µg total protein per sample) were subjected to SDS-PAGE followed by transferring to PVDF membrane. The membrane was incubated for 1 hour in blocking buffer, probed with the primary antibody for 1 hour at room temperature, and then washed twice with blocking buffer without milk. The membrane was then incubated with the appropriate secondary antibody for 1 hour at room temperature. After washing with blocking buffer, the protein bands were visualized using the Western Lightning Plus-ECL Reagent (Perkin Elmer, Waltham, MA, USA). Images of the blots were captured using the BioSpectrumAC Imaging System (UVP, Upland, CA, USA). The band intensity was quantified using UVP Visionworks LS software.

The anti-Endothelin 1 antibody was purchased from Abcam (Cambridge, MA, USA) and the anti-β actin antibody from Sigma-Aldrich (St. Louis, MO, USA). The mouse HRP-conjugated secondary antibody was purchased from Santa Cruz Biotechnology (Santa Cruz, CA, USA).

### Statistical analyses

Statistical analyses of the human tissue microarray, *in situ* hybridization, and qPCR data were performed using the unpaired Student's *t*-test. The cumulative frequency of the pathological changes in the transgenic zebrafish was calculated using Kaplan-Meier analysis [39]. The association of EDN1 and miR-1 expression in patients with HCC was determined using the Wilcoxon signed-rank test [40]. For all of the statistical analyses, a p-value of less than 0.05 was considered to be statistically significant.

## Results

### Generation of Tg(*fabp10a*:*edn1)* transgenic zebrafish

**Figure 1:**
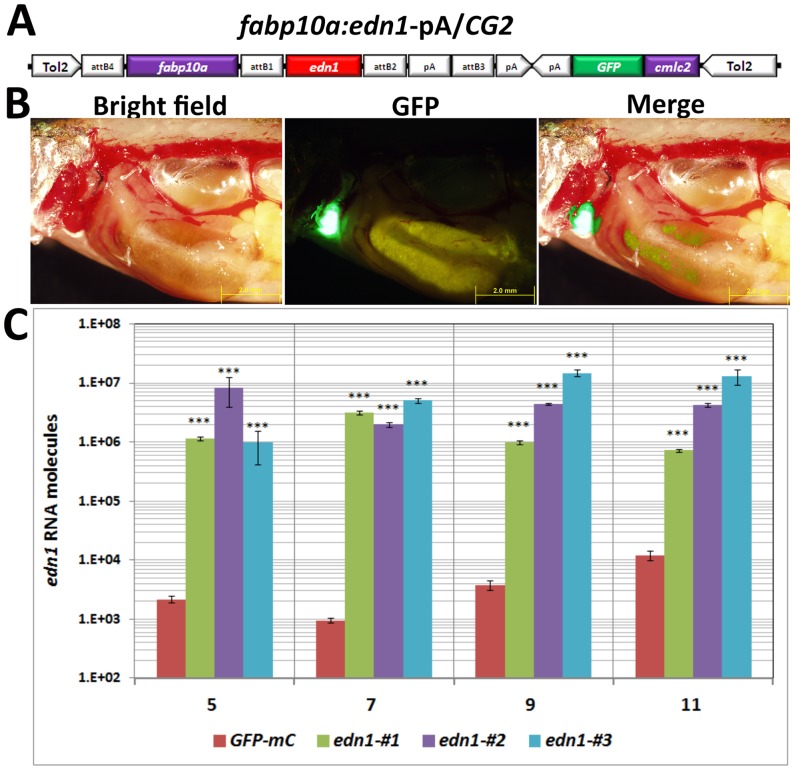
Generation and characterization of *edn1* transgenic fish. (A) Diagram of the *fabp10a*:*edn1*-pA/*CG2* construct that contains Tol2 sequences and the *cmlc2:GFP* expression cassette. (B) The Edn1 protein was expressed in the liver of the wild-type fish carrying the *fabp10a*:*edn1*-pA/*CG2* transgene, as indicated by the expression of *cmlc2*:*GFP* in the heart (green fluorescence, 200X). Scale bars: 2 mm. (C) Quantitative RT-PCR analysis of *edn1* mRNA expression in hepatocytes from 3-, 5-, 7-, 9- and 11-month-old *edn1* transgenic fish (n = 3) and control *fabp10a:GFP-mCherry* transgenic fish. For qPCR analysis, a series of known PCR fragment (GFP) of known concentrations were used as the standard. The *edn1* RNA concentration was calculated base on the standard curve. The differences between the *edn1* transgenic fish and control fish were assessed using a two-tailed Student's *t*-test. Asterisks _***_ represent the P value is less than 0.001.

We previously reported an up-regulation of *Edn1* in the HBx-induced HCC transgenic mouse model using a systems biology approach [28]. To further investigate the role of *edn1* in hepatocarcinogenesis, we generated the pTol2-*fabp10a*:*edn1*-pA/CG2 construct to express *edn1* and *GFP* in zebrafish under the control of the *fabp10a* and *cmlc2* promoter respectively (Fig. 1A). The construct was flanked with the Tol2 transposon element [38] and co-injected with Tol2 transposase mRNA into one-cell embryos to generate *edn1* transgenic zebrafish founder. One transgenic line was generated using a wild-type background zebrafish microinjected with the expression construct, and transgene expression was demonstrated in F1 and F2 fish by visualizing GFP in the heart of 11-month-old fish (Fig. 1B). The expression of *edn1* RNA in three independent Edn1 transgenic fish was assessed by qPCR (Fig. 1C). The absolute amounts of *edn1* RNA by qPCR analysis were previously [37]. More than 1×10^6^ *edn1* RNA molecules from three independent *edn1* transgenic fish were expressed at different stages, but only a few thousand molecules of *edn1* RNA were expressed in the control GFP-mCherry transgenic fish.

### Histopathological examination of the *edn1* transgenic zebrafish

**Figure 2:**
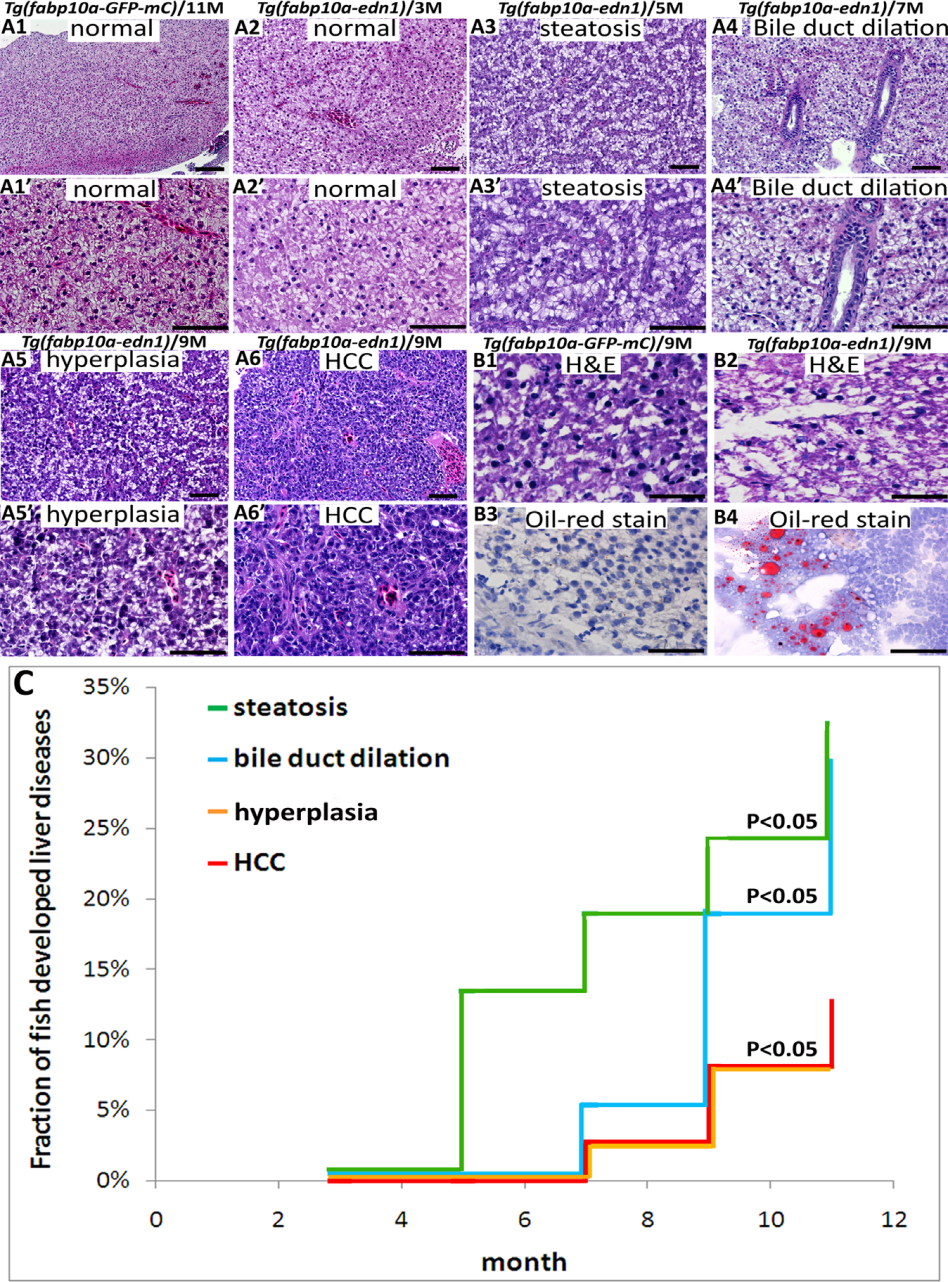
Histopathology of *edn1* transgenic hepatocytes. (A1–A6) H&E staining of the liver sections from the *GFP-mCherry* control fish at 11 months (A1), and from the *edn1* transgenic fish at 3, 5, 7, 9 months (A2–A6). The images were captured at 200X magnification. The A1′–A6′ images resemble the A1–A6 images, though they were captured at 400X magnification. H&E staining of liver sections from *GFP-mCherry* control fish showed normal histology, while *edn1* transgenic fish showed normal, steatosis, bile duct dilation, hyperplasia, and HCC at 3, 5, 7, and 9 months. Scale bars: 50 µm. (B). The Tg(*fabp10a:GFP-mCherry*) and Tg(*fabp10a*:*edn1*-pA/CG2) liver sections were stained by H&E (B1 and B2). The same sections were also stained with oil red O (B3 and B4). Tissues from the Tg(*fabp10a*:*edn1*-pA/CG2) fish showed positive staining with oil red O in the hepatocytes (400X). Scale bars: 50 µm. (C) The cumulative frequency of steatosis, bile duct dilation, hyperplasia, and HCC in the *edn1* transgenic fish was determined by Kaplan-Meier analysis. The different colors denote different liver diseases: steatosis (green), bile duct dilation (blue), hyperplasia (brown), and HCC (red). The differences between the *edn1* transgenic fish and control fish were assessed using a two-tailed Student's *t*-test. The *P* value is less than 0.05.

**Figure 3:**
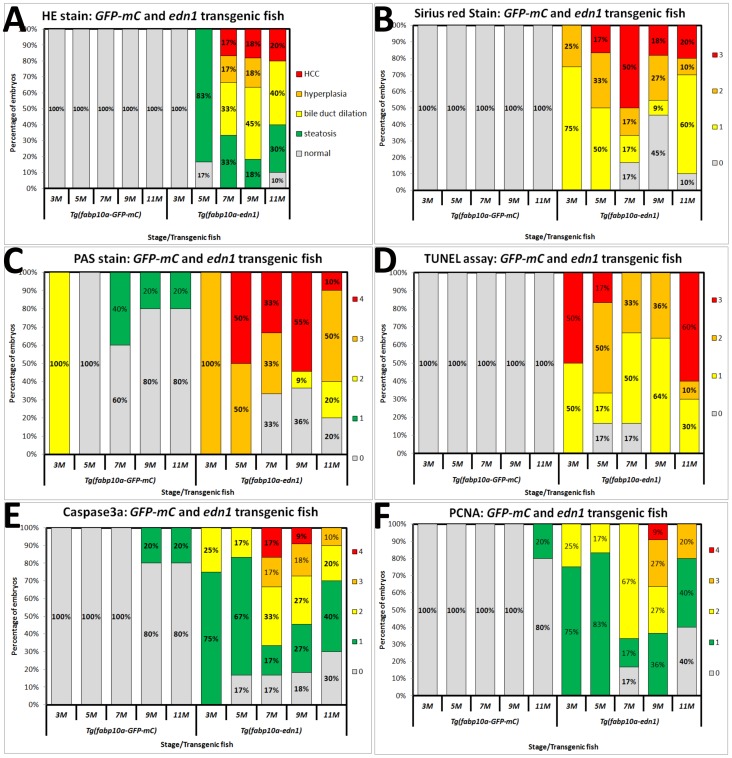
Comparison of hepatocyte histology from *GFP-mCherry* and *edn1* transgenic fish from 3 to 11 months of age. Twenty-seven *GFP-mCherry* transgenic fish (as control) and thirty-seven *edn1* transgenic fish were compared. H&E and additional staining methods were conducted on all the fish samples. (A) Statistical analysis of H&E staining. Different colors denote different pathological features: normal (gray), steatosis (green), bile duct dilation (yellow), hyperplasia (orange), and HCC (red). (B) Liver fibrosis was determined by Sirius Red staining. (C) Glycogen accumulation was identified by Periodic Acid-Schiff (PAS) staining. (D) Apoptosis was examined using the TUNEL assay. (E) Active caspase 3a and (F) Nuclear PCNA expression were assessed by IHC. Different colors represent different pathological scores. There are four possible scores for B and D: 0 (gray), 1 (yellow), 2 (orange), and 3 (red), and five possible scores: 0 (gray), 1 (green), 2 (yellow), 3 (orange), and 4 (red) for C, E and F. The y-axes correspond to “percentage of embryos”.

Liver tissues of the *edn1* transgenic fish derived from the *fabp10a*:*edn1*-pA/CG2 construct and the control transgenic fish from the *fabp10a*:*GFP-mCherry* construct were analyzed at five different ages (3, 5, 7, 9, and 11 months). All of the data presented in this work refer to the fish of the F1 generation. Thirty-seven *edn1* transgenic fish were used for H&E and other staining, and twenty-seven GFP-mCherry transgenic fish were included as control. Detailed results are shown in Table S1. Liver lesions in the zebrafish were examined by an experienced pathologist and were identified based on the criteria developed by the National Toxicology Program and in the literature [41], [42]. Steatosis is characterized by clear intracytoplasmic vacuoles that are positive to the Oil red O staining. Hyperplasia is featured by the accelerated proliferation of atypical hepatocytes with enlarged and mildly irregular nuclei. HCC is identified as an increased nuclear pleomorphism, prominent nucleoli, and an increased number of mitotic figures. The H&E staining revealed that overexpression of *edn1* caused an increase in cytoplasmic vacuoles although the vacuoles were rarely stained red (lipid droplets) by the Oil red O staining (Fig. 2B1–2B4). Additionally, the PAS score showed high levels of glycogen accumulation in the liver with *edn1* overexpression (Fig. 3C). Thus, the cytoplasmic vacuoles may represent both lipid and glycogen accumulation. Overexpression of *edn1* also induced bile duct dilation, hyperplasia, and HCC from five and eleven months of age (Fig. 2A1–2A6). The accumulative frequency of liver pathology was analyzed using the Kaplan-Meier method (Fig. 2C). Compared to the control zebrafish, which showed normal pathology at all stages (Fig. 3A), liver-specific *edn1* expression significantly induced steatosis, bile duct dilation, and HCC (*P*<0.05).

In the control GFP-mCherry fish, all of the liver sections examined were normal based on the H&E staining (from 3 to 11 months of age). However, 83% of the *edn1* transgenic fish showed steatosis by 5 months, and approximately 30% of the fish still exhibited steatosis from 7 to 11 months. Bile duct dilation was observed in 33%–45% of the *edn1* transgenic fish by 7 to 11 months of age. Furthermore, approximately 17–18% of the *edn1* transgenic fish developed hyperplasia by 7 to 9 months. The incidence of HCC formation at 7, 9, and 11 months was 17–20% (Fig. 3A). These results provide the first *in vivo* evidence that *edn1* overexpression in zebrafish facilitates HCC formation.

The penetrance of HCC formation in this study in *edn1* transgenic fish is less than 25%, which is similar to our previous findings that 17% fish showed src-induced HCC [32]. Similarly, overexpression of HBx in p53 mutant fish caused 44% of fish developing HCC at 11 months of age [32]. In a transgenic zebrafish cancer model, overexpression of MYCN resulted in the development of neuroblastoma with a penetrance of less than 20% [39]. Another study demonstrated that overexpression of KrasV12 induced liver tumors with a penetrance of 28% in p53^+/+^ fish, and this rate of tumorigenesis increased to 32% in p53^−/−^ fish [34]. Thus, it is ordinary to observe a low penetrance of tumor formation with overexpression of a single oncogene in the zebrafish animal model, and the synergistic effects between multiple oncogenes can enhance the penetrance of tumor formation.

### Pathological alterations in the livers of *edn1* transgenic zebrafish

We previously demonstrated that liver-specific overexpression of HBx affects collagen expression, glycogen accumulation, apoptosis, and cell proliferation in zebrafish [32], [43]. Thus, in this study we examined these pathological parameters for the *edn1* transgenic fish (Fig. 3). Thirty-seven *edn1* transgenic fish and 27 GFP-mCherry control fish were analyzed using different staining methods as described previously [32]. Representative pictures used for pathological scoring are shown in Fig. S1. In a semi-quantitative analysis using Sirius Red staining, we found that overexpression of *edn1* increased in collagen expression beginning at 3 months and continuing to 11 months while there was no sign of collagen expression in the control fish (Fig. 3B). This result was consistent with a previous study showing that HBx expression in wild-type zebrafish causes fibrosis from 3 to 11 months.

Glycogen accumulation was assessed by Periodic Acid-Schiff (PAS) staining. The results showed that the *edn1* transgenic fish accumulated higher levels of glycogen in the liver compared to the GFP-mCherry control fish (Fig. 3C). Glycogen began to accumulate in the hepatocytes as early as 3 months of age but was decreased with the progression of hepatocarcinogenesis (Fig. 3C). These changes were similar to those observed in the HBx transgenic fish.

Apoptosis was analyzed using the TUNEL assay and immunohistochemical staining for active caspase3. No apoptosis was detected in the control fish, whereas an increased apoptosis was detected in the *edn1* transgenic fish (3 to 11 months of age) (Fig. 3D and 3E). In addition, nuclear PCNA accumulation was observed in the hepatocytes of the *edn1* transgenic fish, and the highest PCNA expression levels was observed at 9 and 11 months. However, only low PCNA expression was observed in the control fish of the same age (Fig. 3F). These pathological alterations reflect the gradual progression of HCC in the *edn1* transgenic zebrafish, from steatosis to fibrosis, hyperplasia, and to HCC within 6 months (between 5 and 11 months of age).

### Up-regulation of genes related to lipid metabolism, cell cycle, and metastasis and increased in fibrosis and tumor markers in *edn1* transgenic zebrafish

**Figure 4:**
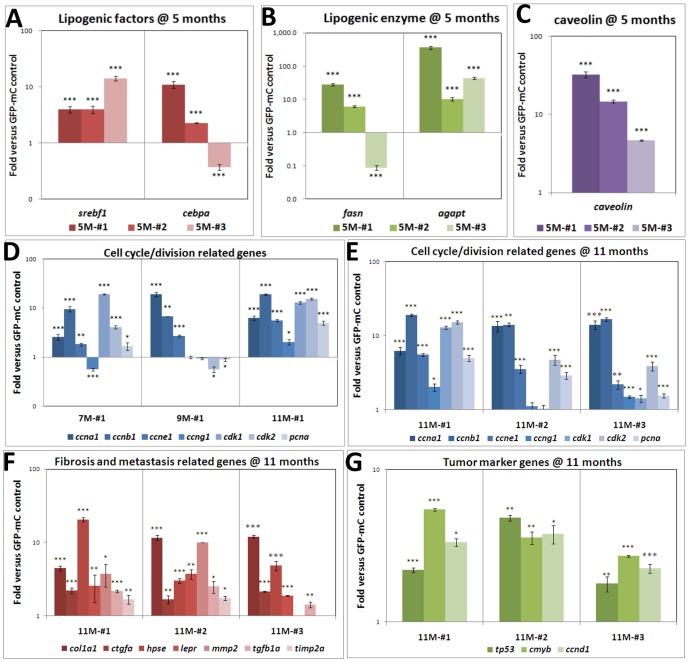
Quantitative RT-PCR analysis of selected marker genes in *edn1* transgenic fish. Genes encoding (A) lipogenic factors, (B) lipogenic enzymes, and (C) PPAR-gamma were up-regulated in 5-month-old *edn1* transgenic fish. (D) Cell cycle/division-related genes were analyzed in fish aged 7 to 11 months and were found to be up-regulated most significantly at 11 months. (E) Cell cycle/division-related genes from three independent *edn1* transgenic fish at 11 month of age were compared. (F) Fibrosis- and metastasis-related genes and (G) tumor markers were up-regulated at 11 months. The mRNA levels of these genes in the liver samples of three independent *edn1* transgenic fish and the age matched *GFP-mCherry* control fish were analyzed by qPCR at 5 different ages (3, 5, 7, 9, and 11 months). Each Ct value was normalized using β-actin as control, and was then compared with the Ct value determined for the *GFP-mCherry* control fish, and then were converted into fold differences. The formula for the relative quantification is: ΔΔCt  =  (C_t,target_ −C_t,β-actin_)_edn1 fish_−(C_t,target_ −C_t, β-actin_)_GFP-mC fish_, and fold change  = 1.94^−ΔΔCt^. Multiple replicates were performed, and the means are presented with their standard deviations. The differences among the variables were assessed using a two-tailed Student's *t*-test. Significant differences between the control and *edn1* transgenic fish are indicated (_*_, *P*<0.05; _**_, *P*<0.01; and _***_, *P*<0.001).

Using quantitative RT-PCR analysis (qPCR), we previously showed that HBx overexpression in wild-type zebrafish enhances expression of several genes related to the observed pathological changes [32]. To assess whether *edn1* overexpression also causes similar effects, we analyzed the expression of lipogenic factors, lipogenic enzymes, the PPAR-γ targeting gene *caveolin*, cell cycle/division related genes, fibrosis and metastasis related genes, and tumor makers. For the qPCR analysis, three biological replicates and multiple technical replicates were performed for each gene at the various stages of the *edn1* transgenic fish and GFP-mCherry control fish. Among these genes, the sterol regulatory binding protein-1 (*srebp1*) gene encodes a transcription factor that binds to the sterol regulatory element-1 (SRE1) site of genes involved in sterol biosynthesis (Fig. 4A). The CCAAT enhancer binding protein-alpha (*cebpa*) gene encodes a bZIP transcription factor that can bind as a homodimer to certain promoters and enhancers and is transcriptionally activated during adipocyte differentiation (Fig. 4A). Fatty acid synthase (FASN) is a multi-enzyme that plays a key role in fatty acid synthesis (Fig. 4B), and acyl-CoA: 1-acylglycerol-sn-3-phosphate acyl-transferase (AGAPT) is an enzyme critical in triglyceride biosynthesis (Fig. 4B). Caveolin belongs to a family of integral membrane proteins that are the principal components of caveolae membranes and are involved in receptor-independent endocytosis (Fig. 4C). All these genes were analyzed in the 5-month-old *edn1* transgenic fish to verify the presence of steatosis at the molecular level.

We also determined the expression levels of cell cycle and cell division related genes, including *ccna1*, *ccnb1*, *ccne1*, *ccng1*, *cdk1*, *cdk2,* and *pcna* in a 7-month-old and a 9-month-old *edn1* transgenic fish (Fig. 4D) as well as in three independent 11-month-old *edn1* transgenic fish (Fig. 4E). Moreover, the fibrosis and metastasis related genes (Fig. 4F) and tumor markers (Fig. 4G) were also analyzed in three independent 11 month-old *edn1* transgenic fish to confirm the presence of hyperplasia and HCC (Fig. 4G). The fibrosis markers examined included 1) collagen of type I alpha 1(*col1a1*); 2) connective tissue growth factor (*ctgfa*), a factor that mirrors some of the effects of TGF beta on skin fibroblasts including the stimulation of extracellular matrix production, chemotaxis, proliferation, and integrin expression; 3) heparanase (*hpse*), an enzyme that acts both at the cell surface and in the extracellular matrix to degrade polymeric heparan sulfate molecules into shorter chain length oligosaccharides; 4) leptin receptor (*lepr*), a single transmembrane domain receptor of the cytokine receptor family that functions as a receptor for the fat cell-specific hormone leptin; 5) matrix metalloproteinase-2 (*mmp2*); 6) TGF-β-1Transforming growth factor beta 1 (*tgfb1a*), a growth factor that acts synergistically with TGFA to induce transformation; and 7) tissue inhibitor of metalloproteinase 2α (*timp2a*), an enzyme that maintains connective tissue integrity by modulating MMP activity. The tumor makers analyzed included 1) protein 53 or tumor protein 53 (*tp53*), a gene that is important in multicellular organisms because of its functions as a tumor suppressor and cell cycle regulator; 2) c-myb proto-oncogene (*cmyb*), a gene important in the control of proliferation and differentiation of hematopoietic progenitor cells; and 3) cyclin D1 (*ccnd1*), a regulatory subunit for CDK4 or CDK6 and an essential factor for the cell cycle G1/S transition. The primer sequences used in qPCR analysis for these genes were listed in Table 1 and also described previously [32].

The qPCR results indicated that the analyzed genes representing lipogenic factors and enzymes and the PPAR-γ targeting caveolin were all up-regulated at 5 months of age in the three independent *edn1* transgenic fish, with the exception that one fish (fish #3) showed a decrease in *cebpa* and *fasn* expression (Fig. 4A, B and C). Many other genes in these categories were examined, and some of them (*pparg*, *chrebp*, *pap*, *dgat2*, *ucp2*, and *cfdl*) showed no significant changes (data not shown). Cell cycle/division-related genes were not up-regulated at 5 months, but showed increases at 7 and 9 months and were markedly increased at 11 months for one of the three *edn1* fish (fish #1) (Fig. 4D). The other two *edn1* transgenic fish also show a similar increasing trend at 11 months, except for the expression of *ccng* and *cdk1* which was not up-regulated in *edn1* fish #2 (Fig. 4E). Moreover, the fibrosis- and metastasis-related genes were also significantly up-regulated in the three *edn1* fish at 11 months with the exception of the TGFb/a expression in *edn1* fish #3 (Fig. 4F). Additionally, the tumor markers – *tp53*, *cmycb,* and *ccnd1* were significantly up-regulated in all three *edn1* transgenic fish compared to the control fish at 11 months (Fig. 4G). Cumulatively, these results demonstrated that the liver-specific expression of *edn1* causes up-regulation of several genes associated with lipid metabolism at 5 months of age, and these changes are consistent with the development of steatosis observed in the pathological analysis. The up-regulation of cell cycle/proliferation-related genes, tumor markers, and metastasis markers detected at 11 months of age in the *edn1* transgenic fish was also in accord with the observation that at this age *edn1* overexpression caused hyperplasia and HCC in zebrafish.

### Screening for *EDN1* expression in multiple normal and cancerous tissue samples

**Figure 5:**
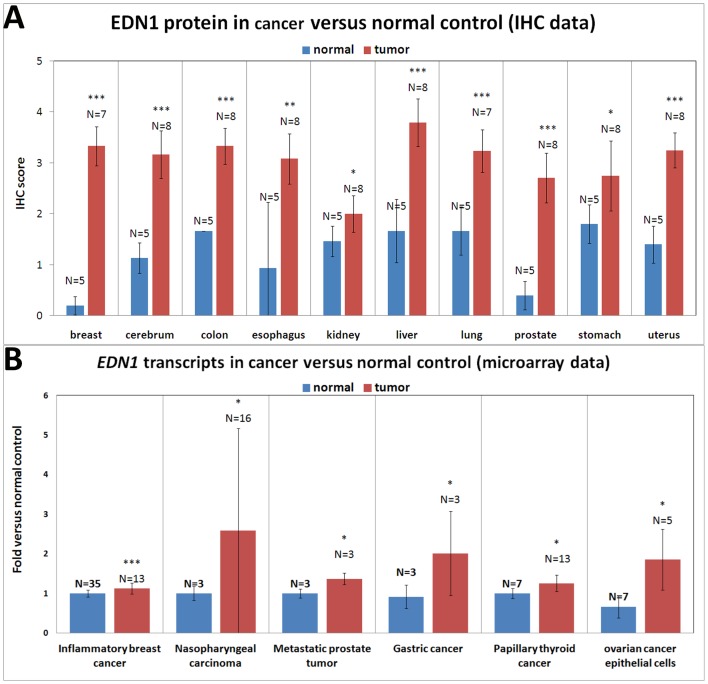
EDN1 protein and mRNA expression in various human tumor and normal tissue samples. (A) To assess EDN1 protein expression in human normal and tumor tissues, the IHC staining of the indicated tissues was performed and the staining intensity was evaluated. A score from 0 to 4 was allocated to each sample and the scores from the same tumor types were averaged. The numbers of samples for each tumor/normal type are indicated. EDN1 expression was enhanced in all tumor types examined. The differences among the variables were assessed using a two-tailed Student's *t*-test. Significant differences between the normal and tumor tissues are indicated (_*_, *P*<0.05; _**_, *P*<0.01; and _***_, *P*<0.001). (B) *EDN1* mRNA expression from the GSE data set. The data of inflammatory breast cancer, nasopharyngeal carcinoma, metastatic prostate tumor, gastric cancer, papillary thyroid cancer, and ovarian cancer were obtained from the GDS3097, GDS3610, GDS2865, GDS1210, GDS1732, and GDS3592 data set, respectively. The numbers of samples for each tumor/normal type are indicated. Over-expression of *EDN1* transcripts was observed in all tumor types examined. The differences among the variables were assessed using a two-tailed Student's *t*-test. Significant differences between the normal and tumor tissues are indicated (_*_, *P*<0.05; _**_, *P*<0.01; and _***_, *P*<0.001).

We previously have shown that *EDN1* expression at the RNA and protein levels was increased in hepatocellular carcinoma samples when compared to normal tissues [28]. To further examine whether the up-regulation of *EDN1* is a common feature in various human cancers, we carried out an immunohistochemical analysis on a cancer tissue array. Additionally, we examined the data set by using the Gene Expression Omnibus website to determine the *EDN1* mRNA levels in tumors and non-tumor samples. *EDN1* overexpression was observed in 80 malignant tumors, which represented 10 tumor types, including breast, cerebrum, colon, liver, lung, prostate, and uterus tumors. EDN1 protein overexpression was less significant in esophagus, kidney and stomach tumors (Fig. 5A). Moreover, comparing with the normal control samples, *EDN1* mRNA expression was increased in inflammatory breast cancer, nasopharyngeal carcinoma, metastatic prostate cancer, gastric cancer, papillary thyroid cancer, and ovarian cancer epithelial cells (Fig. 5B). These results indicated that *EDN1* overexpression is a common phenomenon in the development of various human cancers.

### Stable overexpression of *EDN1* in 293T cells promotes cell proliferation and migration

**Figure 6:**
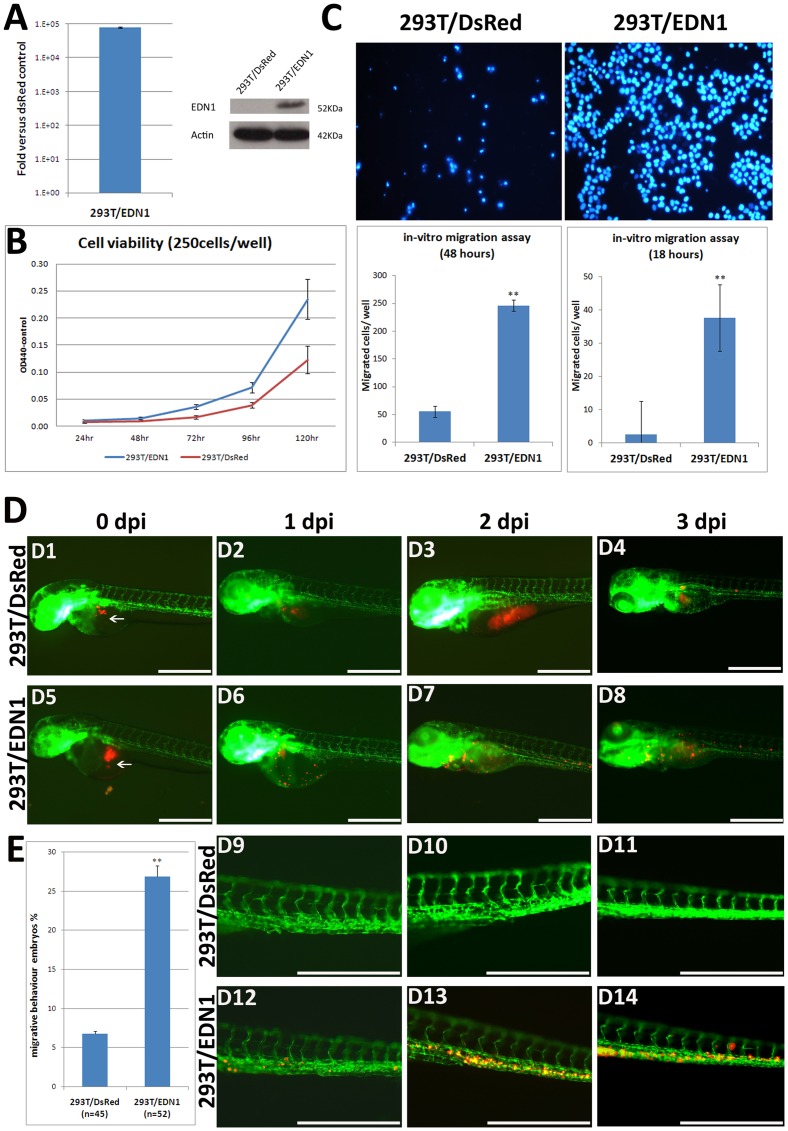
Stable *EDN1* overexpression in 293T cells increases cell viability and migration. (A) *EDN1* RNA and protein expression in cells stably overexpressing *EDN1*. Total RNA was isolated from the 293T/EDN1 and DsRed/293T control cells, and the mRNA expression level was determined by qPCR. Data are expressed as mean ± SD showing the ratio of the *EDN1* expression in the overexpressed cells versus the control cells. The protein expression was analyzed by Western blotting using an EDN1 antibody. The EDN1 protein was expressed exclusively in the stable 293T/EDN1 cells. (B) A WST-1 assay to measure the viability of 293T/EDN1 cells (blue) and 293T/DsRed cells (red); 250 cells/well were seeded in 6-well plates, and the cell viability was measured at 1, 2, 3, 4 and 5 days after seeding. (C) A Transwell assay to assess the migration ability of 293T/EDN1 cells. The method is detailed in the Materials and Methods. The number of migrating 293T/DsRed control cells and 293T/EDN1 cells at 18 and 48 h are expressed as means ± SD of three independent experiment. (D) 293T/DsRed or 293T/EDN1 cells labeled with CM-Dil (red) were ectopically injected into the yolk sac of 2-dpf Tg(*fli1:EGFP*) zebrafish embryos. The *fli1:EGFP* embryos injected with 293T/DsRed control cells (D1–D4) and 293T/EDN1 cells (D5–D8) at 0, 1, 2, and 3 days post-injection (dpi) are shown at a low magnification. The *fli1:EGFP* embryos injected with 293T/DsRed cells (D9–D11) and 293T/EDN1 cells (D12–D14) at 1, 2, or 3 dpi are shown at a high magnification. Arrows indicate the primary injection site. Scale bar: 50 µm. (E) Quantification of the *in-vivo* cell migration. The percentage of the 3 dpi embryos showing migration behavior after xenotransplantation of 293T/DsRed cells (as a control) and 293T/EDN1 cells are shown. Forty-five and thirty-two two-day-old *fli1:EGFP* embryos were injected with DsRed/293T cells and 293T/EDN1 cells, respectively. These cells were examined at 3 dpi for migration behavior with a microscope. The data are presented as the means ± SD. _**_, *P*<0.001.

To further elucidate the molecular mechanism by which *edn1* overexpression induces hepatocarcinogenesis, we used a HEK-293T cell line to overexpress *EDN1*. HEK-293T cells were used because of the ease of culturing and their high transfection efficiency. These cells were derived from human embryonic kidney cells and are widely used in laboratory research. HEK-293 cell lines with high passages (>65 passages) are 100% tumorigenic, whereas low passage cells (<52 passages) exhibit no tumorgenecity under the same culturing conditions [44]. For the purpose of this study, low passage HEK-293T cells (passage 15) were used to generate the *EDN1* stably expressed cell lines. As shown in Fig. 6, the stable line obtained significantly enhanced the *EDN1* RNA and protein levels (Fig. 6A) as well as the cell viability at low densities as analyzed by the WST-1 assay (Fig. 6B). *EDN1* overexpression also enhanced *in-vitro* migration by more than five folds compared to the control cells at both 18 hours and 48 hours after seeding (Fig. 6C), in which 18-h incubation exhibited a stronger effect than the 48-h incubation. Although *EDN1* overexpression could increase cell proliferation, the “migrated cells” observed in the 18-h incubation are not a result of increased proliferation because this incubation time is less than the time required for cell doubling.

To evaluate *in-vivo* migration, we employed a xenotransplantation assay, in which 293T/DsRed cells as control and 293T/EDN1 cells were labeled with DiI and injected into 2 day-old *fli1:EGFP* zebrafish embryos. At three days post-injection (dpi) these embryos were assayed for migration under a fluorescence microscope (Fig. 6D). Embryos that lacked fluorescence after injection or contained migrated cells 2 hours post-injection (indicating cell leakage) were eliminated from the analysis. These results showed that the embryos injected with the control 293T/DsRed cells remained the fluorescence in the yolk up to 3 dpi (Fig. 6D1–D4 and D9–D11), while the embryos injected with 293T/EDN1 cells showed the cells migrated into the entire trunk and tail (Fig. 6D5–D8 and D12–D14). In few embryos, we observed the distant migration and extravasation of these cells further within the trunk. The embryos showing migration behavior after injection of 293T/EDN1 cells and 293T/DsRed cells accounted for 26.9% and 6.8% of the fish respectively (Fig. 6E).

### *EDN1* overexpression results in an up-regulation of genes related to the cell cycle, migration, and the UPR pathway, and EDN1 inhibitor and AKT inhibitor can reverse these changes

**Figure 7:**
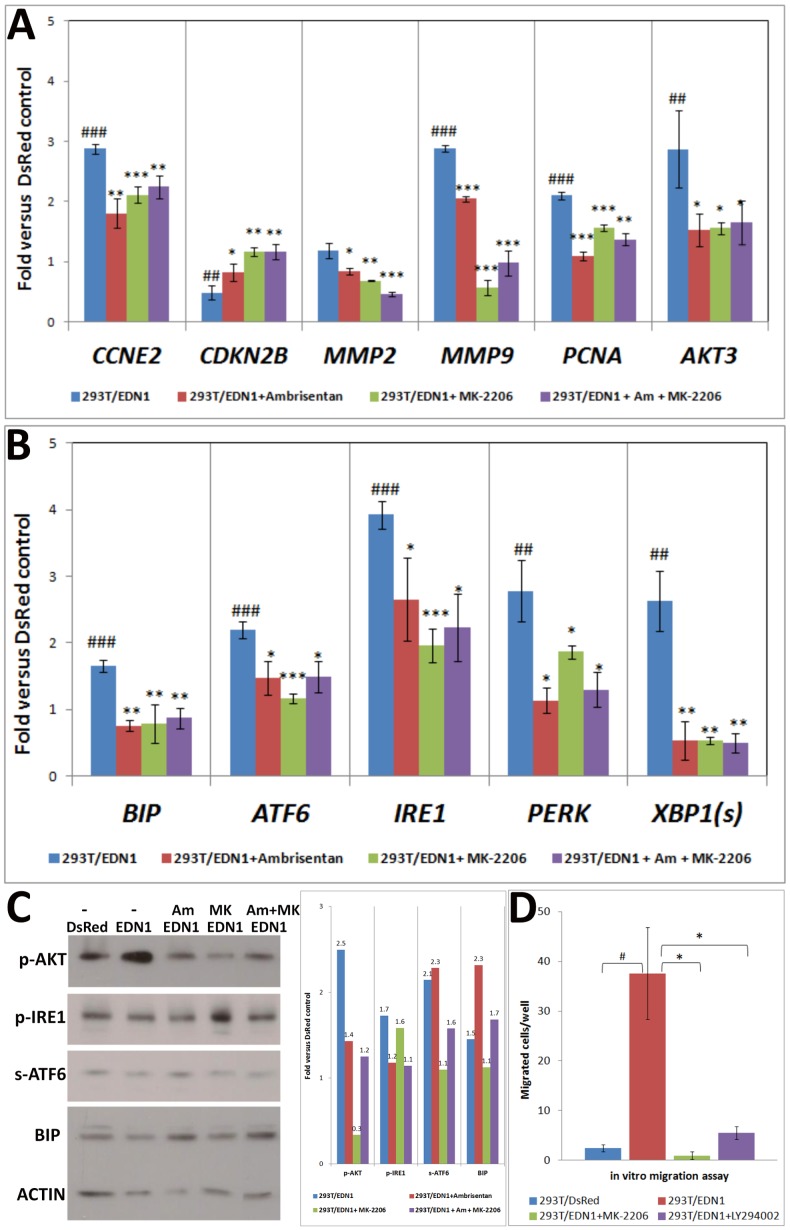
Quantitative RT-PCR analysis of selected marker genes in cells stably overexpressing *EDN1*. 293T/EDN1 and 293T/DsRed stable cells at passage 15 and 18, respectively were used. Data are the means ± SD of three to seven independent experiments. Statistical significance was determined using Student's t-test. (A–B) RNA expression levels of the cell cycle-, proliferation-, and migration-related genes (A) and the mediators of the UPR pathway (B) in the EDN1 stable cells cultured in the absent (blue) or presence of the EDN1 inhibitor Ambrisentan (red); the AKT inhibitor MK-2206 (green), or the combination of the two inhibitors (purple). (C) Western blots and the quantification of the signal intensities. The expression of the UPR mediators (p-AKT, p-IRE1, proteolytic ATF6, and BIP) was detected in the stable EDN1 cells incubated in the absence (blue) or presence of the EDN1 inhibitor Ambrisentan (red); the AKT inhibitor MK-2206 (green), or combination of the two inhibitors (purple). (D) *in vitro* migration assay of the DsRed control cells (darkest blue) and the stable EDN1 cells incubated in the absence (dark blue) or presence of the AKT inhibitor MK-2206 (light blue) or the PI3K inhibitor LY294002 (lightest blue). Significant differences among the groups are indicated (_*_, *P*<0.05; _**_, *P*<0.01; _***_, *P*<0.001).

To understand the molecular mechanism underlying the *EDN1*-induced proliferation and migration, we measured the expression levels of the genes related to cell cycle, proliferation, and migration by qPCR. The 293T cells overexpressing the DsRed vector were used as a control. An up-regulation of the *CCNE2*, *CDK2b*, *MMP2*, *MMP9*, *PCNA,* and *AKT3* genes was observed in the 293T cells overexpressing *EDN1* (Fig. 7A). We also determined the expression levels of the mediators of the UPR pathway as this pathway is associated with the risk of steatosis, glycogen accumulation, and tumor formation, all these conditions being observed in the *edn1* transgenic fish. By qPCR analysis, we found that spliced *XBP1, ATF6*, *BIP*, *IRE1,* and *PERK* were up-regulated in the 293T/EDN1 cells compared to the control cells (Fig. 7B). It has been shown that EDN1 mediates the activation of the PI3K/AKT pathway in lung fibrogenesis and in human chondrosarcoma cells [45], [46]. Thus, to assess whether the up-regulation of these genes also is mediated by AKT activation, we used the EDN1 and AKT inhibitors Ambrisentan and MK-2206, respectively, to treat the 293T stable cells. The control 293T/DsRed cells incubated with Ambrisentan and/or MK-2206 had no significant changes in the RNA levels of the genes tested (Fig. S2). However, the presence of Ambrisentan or MK-2206, the increase in the RNA expression of these genes in the 293T/EDN1 cells was reversed to the levels not different from the 293T/DsRed control cells (Figs. 7A–B). These results confirmed the hypothesis that by binding to its receptor, EDN1 activates the PI3K/AKT signal pathway and then induces the expression of the downstream target genes, including those related to cell cycle, migration, and the UPR pathway. Because the UPR pathway-related genes are also targets of the EDN1/PI3K/AKT pathway, this might partially explain the conditions of steatosis, glycogen accumulation, fibrosis, and HCC observed in the HBx and *edn1* transgenic fish.

We also performed western blot analysis to determine the protein levels of phospho-AKT, phospho-IRE1, proteolytic ATF6, and BIP, all being the effectors of the UPR pathway. As shown in Fig. 7C, AKT phosphorylation was increased in 293T/EDN1 cells compared to the 293T/DsRed control cells, and it was blocked in the presence of Ambrisentan or MK-2206. The EDN1-induced IRE1 phosphorylation also was reduced by Ambrisentan. The up-regulation of the proteolytic ATF6 and BIP expression following EDN1 overexpression was diminished in the presence of MK-2206. These results confirmed that the UPR mediators were increased by EDN1 at the protein level and that these changes can be reversed by AKT and EDN1 inhibitors. Using the same inhibitors, we further demonstrated that inhibition of AKT or PI3K also blocked the EDN1-induced cell migration (Fig. 7D). This finding provides additional evidence for a link between EDN1, PI3/AKT, and cell migration ability.

**Figure 8:**
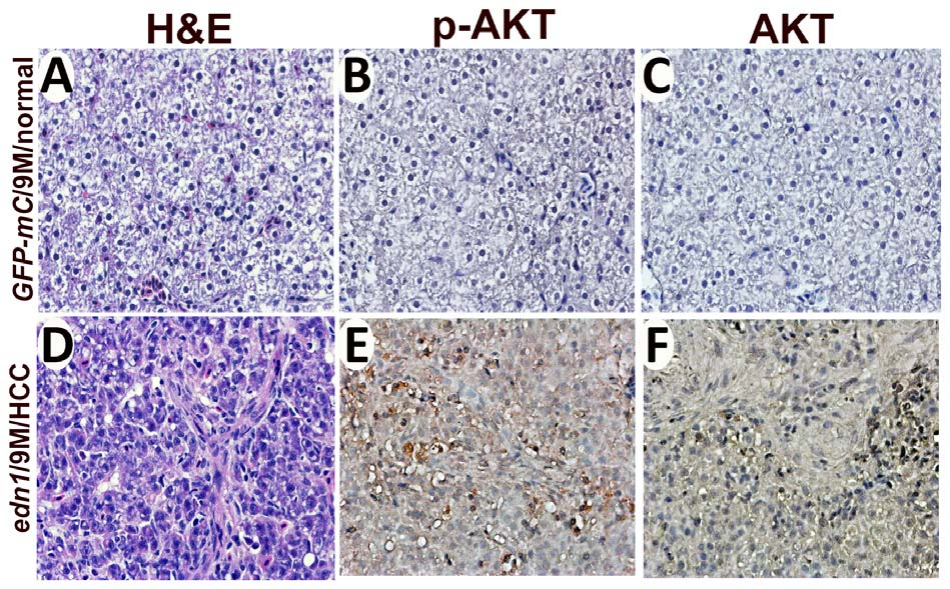
Expression of p-AKT and AKT in the *edn1* transgenic fish. (A) and (D) depict H&E staining of the liver sections of the GFP-mCherry control and *edn1* transgenic fish. (B-C) and (E-F) shows the IHC staining using an anti-phospho-AKT antibody (B and E) or an anti-AKT antibody (C and F).

The involvement of EDN1 in the AKT pathway was also explored using the *edn1* transgenic fish. By immunostaining with antibodies specific to phospho-AKT (p-AKT) and AKT, we found that the p-AKT staining is enhanced in the *edn1* transgenic fish compared to the GFP-mCherry control fish (Fig. 8B vs. 8E), while the AKT level was not significantly different between the two transgenic fish (Fig. 8C vs. 8F). We observed hyperplasia and HCC in *edn1* transgenic fish, which mimics hepatocyte proliferation in humans. These data may support the hypothesis that EDN1 promotes liver cell proliferation through the AKT pathway.

### Association of EDN1 and miR-1 expression in patients with HCC

**Figure 9:**
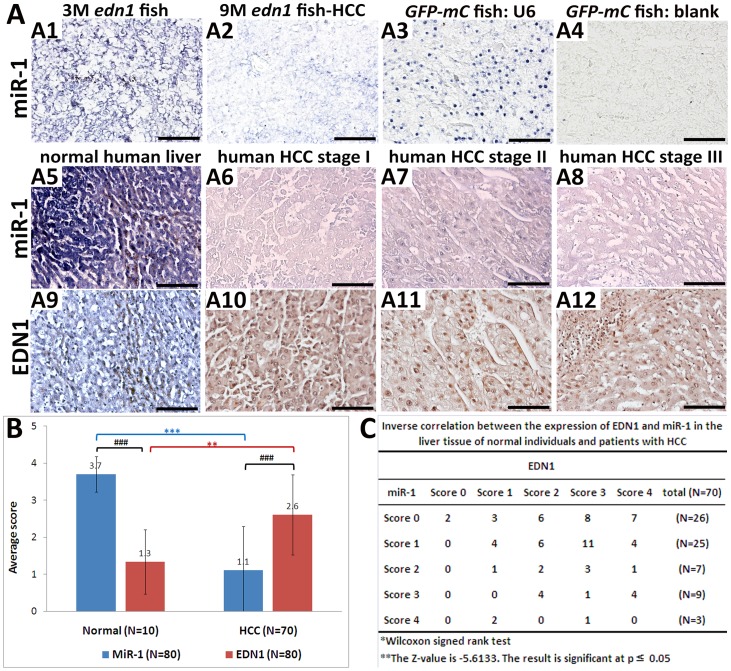
MiR-1 regulates EDN1 expression in HCC. (A) The RNA expression of miR-1 in the *edn1* transgenic fish and human specimens was determined by *in-situ* hybridization, and EDN1 protein expression in human specimen by IHC. The expression of miR-1 was at the high level in 3-month-old *edn1* transgenic fish which is normal in pathological analysis (A1) and was decreased in the 9-month-old *edn1* transgenic fish with HCC (A2). The liver tissue of the GFP-mCherry control zebrafish was stained with (A3) or without (A4) the U6 probe as the positive or negative control, respectively. The miR-1 signal was very intense in the normal human liver tissues (A5), but was weakened in the tissue of HCC at stage I to III tissues. (A6–8). The EDN1 signal was very weak in the normal liver tissue (A9), but became intense in the tissue of HCC at stage I to III (A10–12). The positive signal is shown in purple for miR-1 *in-situ* and brown for EDN1 IHC (200X). Scale bars: 50 µm. (B) Comparison of the expression of EDN1 and miR-1 in normal and HCC tissues. The staining intensity was scored from 0 to 4. _** or ##_, *P*<0.001; _*** or ###_, *P*<0.0001. (C) Correlation between EDN1 and miR-1 expression in the hepatic tissue of HCC patients.

Several *in vitro* studies have indicated that the 3′ untranslated region (3′UTR) of *EDN1* is a direct target of miR-1, and hence miR-1 inhibit the *EDN1* expression and lead to attenuation of cell proliferation [28]. To assess the relation of miR-1 to *EDN1*, we initially measured the expression level of miR-1 in the *edn1* transgenic fish. As shown in Fig. 9A, miR-1 was expressed at high levels in the 3-month-old *edn1* transgenic fish (Fig. 9A1), but the expression was reduced in the 9-month-old *edn1* transgenic fish with HCC (Fig. 9A2). Thus, the miR-1 level is inversely related to the development of HCC. Because the *edn1* gene in the *edn1* transgenic fish does not include the 3′ UTR, the *edn1* expression in the fish should not be targeted by miR-1. Moreover, the *edn1* mRNA levels were at high levels at all ages of the transgenic fish. Interestingly, the miR-1 levels were decreased in the HCC samples of the 9 month-old fish compared to those in the normal tissue samples of the 3 month-old *edn1* transgenic fish, suggesting that miR-1 may be negatively regulated by *edn1*.

Using the immunohistochemistry and in-situ hybridization approaches, we further evaluated the relationship between the EDN1 protein and miR-1 RNA levels in human HCC tissues. The results showed that the expression of miR-1 was significantly higher in normal liver tissue (Fig. 9A5) than in the HCC specimens at stages I to III (Figs. 9A6–A8). Conversely, the EDN1 protein expression was significantly lower in the normal tissue (Fig. 9A9) than in the HCC tissues (Figs. 9A10–A12). Statistical analysis demonstrated that there was an inverse correlation between the EDN1 and miR-1 levels in the HCC tissues (Figs. 9B–C). These results suggested that up-regulation of EDN1 in HCC may be caused by a down-regulation of miR-1 in these patients.

## Discussion

The development of liver cancer is a multistep process that includes inflammation, fibrosis, cirrhosis, and finally, HCC. In Asia, more than 80% of HCC cases are due to infection with HBV or HCV. In this study, a zebrafish model was used to study the molecular mechanism of hepatocarcinogenesis, and can also serve as a high-throughput drug-screening platform. Many oncogenes, such as gankyrin, HBx, YY1, KrasV12, Xmrk, and Myc, have been shown to induce steatosis or HCC in zebrafish, and the tumors induced share many similar characteristics with human tumors [32]–[36], [47]–[49]. Herein, we report that the liver-specific expression of *edn1* causes steatosis, fibrosis, hyperplasia, and HCC in the *edn1* transgenic zebrafish. In addition, glycogen accumulation also was increased in these fish. The HCC formation was analyzed by histological, immunochemical and molecular biology methods, and the immunochemical data indicated an increase in proliferating cell nuclear antigen (PCNA) and active caspase 3 expression in the *edn1* transgenic fish at 7 to 11 months of age, which correlates with the age of hyperplasia and HCC formation.

EDN1 is a growth-promoting peptide that stimulates the proliferation of many malignant cells, such as melanoma, hepatoma, prostate, colorectal, and ovarian cancer cells. We previously have demonstrated that *Edn1* is significantly up-regulated in the HBx-induced HCC model in mice [28]. In this study, we also detected the increase in EDN1 protein expression in breast, cerebrum, liver, and prostate tumors. Those findings indicate a role of EDN1 in the cancer development. Using HEK-293T cells, we observed that overexpression of EDN1 enhanced cell proliferation *in vitro* and cell migration both *in vitro* and *in vivo*, and these functional changes might be triggered by the EDN1-induced expression of cell cycle-, proliferation- and migration-related genes. Moreover, the up-regulation of these target genes may be mediated by activation of the AKT signal pathway, as inferred by the reversal effects of EDN1 and AKT inhibitors. Several genes related to the UPR pathway also appeared to be the downstream targets of the EDN1/PI3K/AKT pathway. It has been reported that ER stress negatively regulates the expression of the miR-199a/214 cluster in HCC and is required for tumor survival and progression [50]. Additionally, the UPR mediator ATF6-alpha was found to be involved in hepatocarcinogenesis [51]. Our data indicated that, EDN1 and the UPR can be linked through the AKT pathway, which is known to be involved in cell proliferation and migration, and this EDN1/AKT/UPR link might be the mechanism underlying the development of hepatocarcinogenesis in the *edn1* transgenic zebrafish.

miRNAs regulate gene expression by inhibiting translation or by degrading target mRNA and are involved in liver cancer progression. microRNA-1 (miR-1) inhibits cell proliferation in HCC by targeting EDN1 [28]. miR-1 is abnormally down-regulated in several types of cancers, including lung, colorectal, prostate, and thyroid cancers and rhabdomyosarcoma. It acts as a tumor suppressor and enables to inhibit cell proliferation and promote cell differentiation and apoptosis [28]. In this study, we found that miR-1 was significantly down-regulated and EDN1 was up-regulated in HCC tissues. These findings indicated a potential role of miR-1 in EDN1 regulation HCC.

In conclusion, we report that the liver-specific expression of *edn1* induced hepatocarcinogenesis in zebrafish. The overexpression of EDN1 in HEK-293T cells enhanced cell proliferation and migration. Many genes involved in cell-cycle, migration, and the UPR pathway were up-regulated in the cells stably overexpressing EDN1. The up-regulation of these genes was reversed by EDN1 and AKT inhibitors. Finally, we are the first to report that the up-regulation of EDN1 in HCC is correlated with a down-regulation of miR-1. Notably, the correlation between EDN1 and miR-1 was observed in the tissues of HCC patients, highlighting the clinical significance of our observations. Thus, our findings provide a novel mechanism for the regulation of EDN1 in HCC, which advances our understanding on both the pleiotropic nature of the EDN1 protein and its role in HCC formation. Moreover, gene therapy using miRNA mimics may be useful for the treatment of HCC. Our zebrafish model also provides new possibilities for screening potential therapeutics to prevent the development and progression of HCC.

## Supporting Information

Figure S1 **Representative images from different staining methods and their scoring standards.** (A) Sirius Red staining (200X), (B) TUNEL assay (200X), (C) PAS staining (200X), (D) caspase 3 staining (400X), and (E) nuclear PCNA staining (400X). Scale bar: 50 µm.
(TIF)

Figure S2 **Effects of EDN1 and AKT inhibitors on gene expression in DsRed/293T cell.** RNA was isolated from the DsRed/293T cells cultured in the absence or presence of the EDN1 inhibitor Ambrisentan, the AKT inhibitor MK-2206, or with the combination of the two inhibitors. Expression of the cell cycle- and proliferation-related genes and UPR mediators were analyzed by qPCR.
(TIF)

Table S1 **Summary of the H&E staining results showing the pathological changes of liver tumor progression in** ***GFP-mCherry*** **and** ***edn1*** **transgenic fish.**
(DOC)
