# Supplementary figures and images for: Overexpression of Endothelin 1 Triggers Hepatocarcinogenesis in Zebrafish and Promotes Cell Proliferation and Migration through the AKT Pathway
Source: PLoS One. 2014 Jan 8;9(1):e85318. doi: 10.1371/journal.pone.0085318 (PMC3885696; doi:10.1371/journal.pone.0085318)

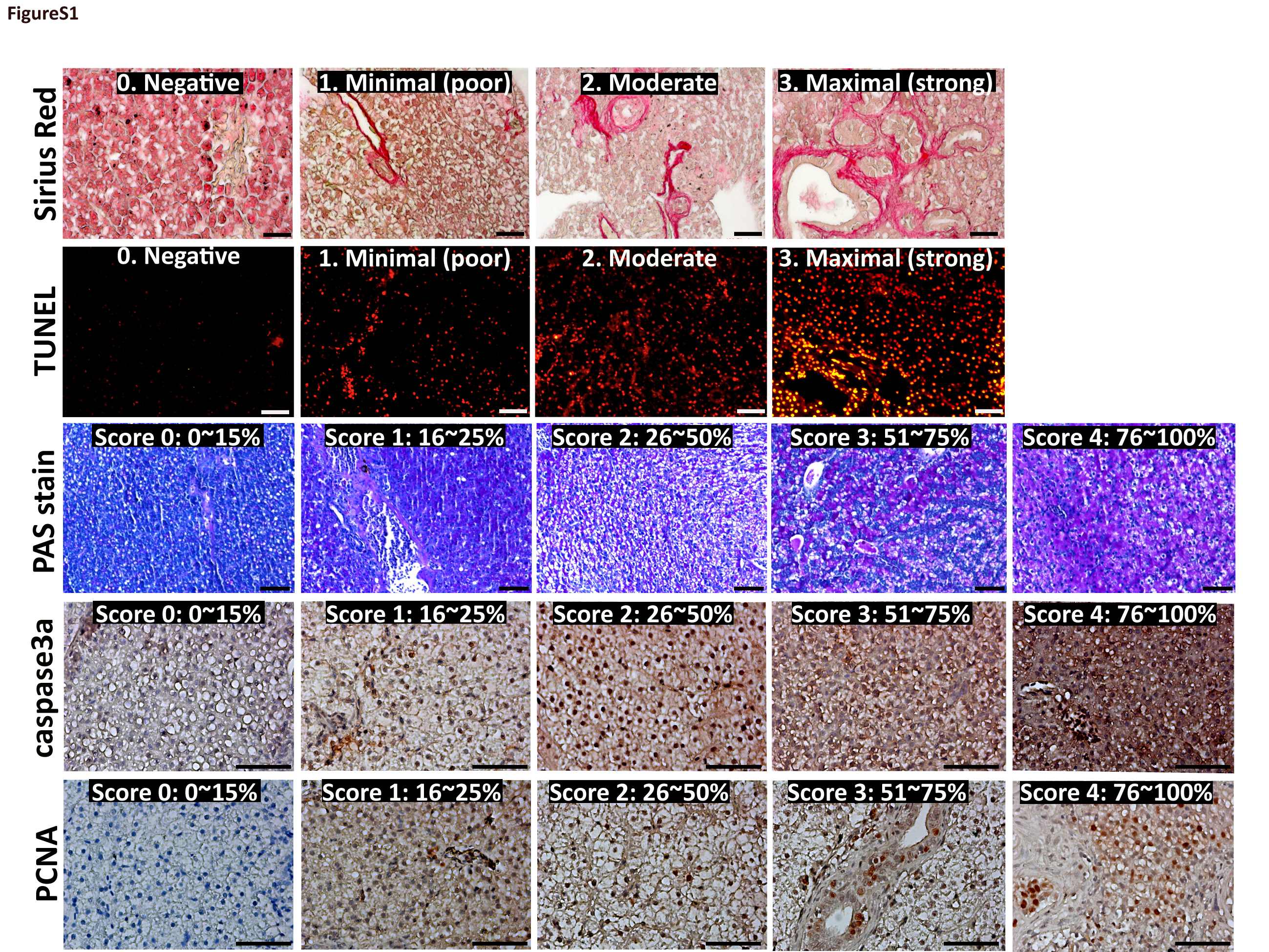

Supplement: Figure S1 — Representative images from different staining methods and their scoring standards. (A) Sirius Red staining (200X), (B) TUNEL assay (200X), (C) PAS staining (200X), (D) caspase 3 staining (400X), and (E) nuclear PCNA staining (400X). Scale bar: 50 µm. (TIF) [file pone.0085318.s001.tif]

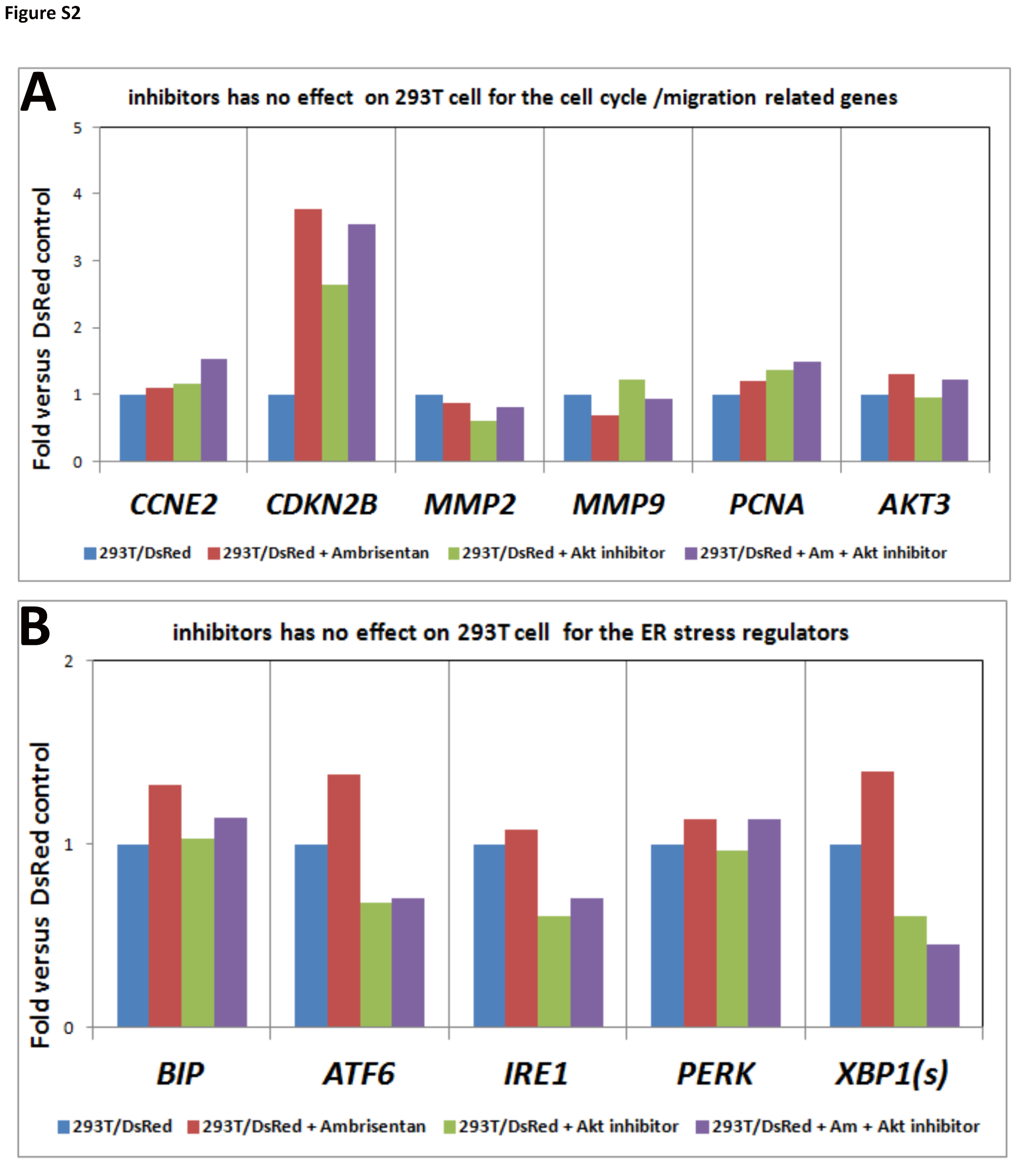

Supplement: Figure S2 — Effects of EDN1 and AKT inhibitors on gene expression in DsRed/293T cell. RNA was isolated from the DsRed/293T cells cultured in the absence or presence of the EDN1 inhibitor Ambrisentan, the AKT inhibitor MK-2206, or with the combination of the two inhibitors. Expression of the cell cycle- and proliferation-related genes and UPR mediators were analyzed by qPCR. (TIF) [file pone.0085318.s002.tif]
