# Supplementary material for: Overexpression of Endothelin 1 Triggers Hepatocarcinogenesis in Zebrafish and Promotes Cell Proliferation and Migration through the AKT Pathway
Source: PLoS One. 2014 Jan 8;9(1):e85318. doi: 10.1371/journal.pone.0085318 (PMC3885696; doi:10.1371/journal.pone.0085318)
Supplement: Table S1 — Summary of the H&E staining results showing the pathological changes of liver tumor progression in GFP-mCherry and edn1 transgenic fish. (DOC) [file pone.0085318.s003.doc]

**Table S1.** Summary of H&E staining revealed the pathology of liver tumor progression in *GFP-mCherry* control and *edn1* transgenic fish

| Number(N=19) | Gender | Months | Type | H&E (Diagnosis) | Sirius red (Score) | PAS (Score) | TUNEL (Score) | Caspase3a (Score) | PCNA (Score) |
| --- | --- | --- | --- | --- | --- | --- | --- | --- | --- |
| 1 |  | 3 | Control | Normal | 0 | 2 | 0 | 0 | 0 |
| 2 |  | 5 | Control | Normal | 0 | 0 | 0 | 0 | 0 |
| 3 |  | 5 | Control | Normal | 0 | 0 | 0 | 0 | 0 |
| 4 |  | 5 | Control | Normal | 0 | 0 | 0 | 0 | 0 |
| 5 | F | 7 | Control | Normal | 0 | 0 | 0 | 0 | 0 |
| 6 | M | 7 | Control | Normal | 0 | 1 | 0 | 0 | 0 |
| 7 | M | 7 | Control | Normal | 0 | 0 | 0 | 0 | 0 |
| 8 | M | 7 | Control | Normal | 0 | 1 | 0 | 0 | 0 |
| 9 | M | 7 | Control | Normal | 0 | 0 | 0 | 0 | 0 |
| 10 | M | 9 | Control | Normal | 0 | 1 | 0 | 0 | 0 |
| 11 | M | 9 | Control | Normal | 0 | 0 | 0 | 0 | 0 |
| 12 | M | 9 | Control | Normal | 0 | 0 | 0 | 0 | 0 |
| 13 | F | 9 | Control | Normal | 0 | 0 | 0 | 1 | 0 |
| 14 | F | 9 | Control | Normal | 0 | 0 | 0 | 0 | 0 |
| 15 | F | 11 | Control | Normal | 0 | 0 | 0 | 0 | 0 |
| 16 | F | 11 | Control | Normal | 0 | 0 | 0 | 0 | 0 |
| 17 | M | 11 | Control | Normal | 0 | 0 | 0 | 0 | 1 |
| 18 | M | 11 | Control | Normal | 0 | 1 | 0 | 1 | 0 |
| 19 | M | 11 | Control | Normal | 0 | 0 | 0 | 0 | 0 |
| Number(N=37) | Gender | Months | Type | H&E (Diagnosis) | Sirius red (Score) | PAS (Score) | TUNEL (Score) | Caspase3a (Score) | PCNA (Score) |
| 1 | M | 3 | *edn1* | Normal | 1 | 3 | 1 | 1 | 1 |
| 2 | F | 3 | *edn1* | Normal | 1 | 3 | 3 | 1 | 2 |
| 3 | M | 3 | *edn1* | Normal | 1 | 3 | 1 | 2 | 1 |
| 4 | M | 3 | *edn1* | Normal | 2 | 3 | 3 | 1 | 1 |
| 5 | M | 5 | *edn1* | Steatosis | 2 | 4 | 2 | 0 | 1 |
| 6 | M | 5 | *edn1* | Steatosis | 3 | 4 | 2 | 1 | 1 |
| 7 | M | 5 | *edn1* | Steatosis | 2 | 4 | 1 | 1 | 1 |
| 8 | M | 5 | *edn1* | Steatosis | 1 | 3 | 3 | 1 | 1 |
| 9 | M | 5 | *edn1* | Normal | 1 | 3 | 2 | 1 | 1 |
| 10 | F | 5 | *edn1* | Steatosis | 1 | 3 | 0 | 2 | 2 |
| 11 | M | 7 | *edn1* | Steatosis | 1 | 3 | 1 | 3 | 2 |
| 12 | F | 7 | *edn1* | Hyperplasia | 3 | 0 | 1 | 0 | 2 |
| 13 | M | 7 | *edn1* | Bile duct dilation | 2 | 4 | 2 | 1 | 0 |
| 14 | M | 7 | *edn1* | Bile duct dilation | 3 | 4 | 2 | 2 | 2 |
| 15 | M | 7 | *edn1* | Steatosis | 3 | 3 | 1 | 2 | 2 |
| 16 | F | 7 | *edn1* | HCC | 0 | 0 | 0 | 4 | 1 |
| 17 | F | 9 | *edn1* | HCC | 0 | 0 | 1 | 3 | 1 |
| 18 | F | 9 | *edn1* | Hyperplasia | 0 | 0 | 1 | 3 | 1 |
| 19 | M | 9 | *edn1* | Steatosis | 0 | 4 | 2 | 0 | 1 |
| 20 | M | 9 | *edn1* | Bile duct dilation | 3 | 4 | 2 | 0 | 3 |
| 21 | M | 9 | *edn1* | Bile duct dilation | 1 | 4 | 1 | 2 | 3 |
| 22 | M | 9 | *edn1* | HCC | 0 | 0 | 1 | 2 | 4 |
| 23 | M | 9 | *edn1* | Bile duct dilation | 3 | 4 | 1 | 1 | 2 |
| 24 | M | 9 | *edn1* | Bile duct dilation | 2 | 2 | 2 | 1 | 2 |
| 25 | M | 9 | *edn1* | Hyperplasia | 0 | 0 | 1 | 4 | 1 |
| 26 | M | 9 | *edn1* | Bile duct dilation | 2 | 4 | 1 | 2 | 3 |
| 27 | M | 9 | *edn1* | Steatosis | 2 | 4 | 2 | 1 | 2 |
| 28 | F | 11 | *edn1* | HCC | 1 | 0 | 2 | 0 | 0 |
| 29 | F | 11 | *edn1* | HCC | 1 | 0 | 1 | 0 | 0 |
| 30 | M | 11 | *edn1* | Steatosis | 1 | 4 | 1 | 0 | 1 |
| 31 | M | 11 | *edn1* | Steatosis | 0 | 3 | 1 | 1 | 1 |
| 32 | M | 11 | *edn1* | Bile duct dilation | 1 | 3 | 3 | 1 | 0 |
| 33 | M | 11 | *edn1* | Steatosis | 1 | 3 | 3 | 1 | 0 |
| 34 | M | 11 | *edn1* | Bile duct dilation | 2 | 3 | 3 | 1 | 3 |
| 35 | M | 11 | *edn1* | Bile duct dilation | 3 | 3 | 3 | 2 | 3 |
| 36 | M | 11 | *edn1* | Normal | 3 | 2 | 3 | 2 | 1 |
| 37 | M | 11 | *edn1* | Bile duct dilation | 1 | 2 | 3 | 3 | 1 |
